# Crystal structure and Hirshfeld surface analysis of (*E*)-1-[2,2-di­bromo-1-(4-nitro­phen­yl)ethen­yl]-2-(4-fluoro­phen­yl)diazene

**DOI:** 10.1107/S2056989022004388

**Published:** 2022-04-28

**Authors:** Zeliha Atioğlu, Mehmet Akkurt, Namiq Q. Shikhaliyev, Naila A. Mammadova, Gulnara V. Babayeva, Victor N. Khrustalev, Ajaya Bhattarai

**Affiliations:** a Department of Aircraft Electrics and Electronics, School of Applied Sciences, Cappadocia University, Mustafapaşa, 50420 Ürgüp, Nevşehir, Turkey; b Department of Physics, Faculty of Sciences, Erciyes University, 38039 Kayseri, Turkey; c Organic Chemistry Department, Baku State University, Z. Khalilov str. 23, AZ 1148 Baku, Azerbaijan; d Azerbaijan State Pedagogical University, Uzeyir Hajibeyli str., 68, Baku, Azerbaijan; e Peoples’ Friendship University of Russia, 6 Miklukho-Maklaya, Moscow, Russian Federation; f N.D. Zelinsky Institute of Organic Chemistry, Russian Academy of Sciences, 47 Leninsky Av., Moscow, Russian Federation; g Department of Chemistry, M.M.A.M.C (Tribhuvan University) Biratnagar, Nepal

**Keywords:** crystal structure, C—H⋯O hydrogen bonds, C—H⋯F hydrogen bonds, C—Br⋯π inter­actions, C—F⋯π inter­actions, π–π stacking inter­actions, Hirshfeld surface analysis

## Abstract

In the title compound, C_14_H_8_Br_2_FN_3_O_2_, the 4-fluoro­phenyl ring and the nitro-substituted phenyl ring form a dihedral angle of 64.37 (10)°. Mol­ecules in the crystal are connected by C—H⋯O and C—H⋯F hydrogen bonds into layers parallel to (011). The crystal packing is consolidated by C—Br⋯π and C—F⋯π inter­actions, as well as by π–π stacking inter­actions. According to a Hirshfeld surface analysis of the crystal structure, the most significant contributions to the crystal packing are from O⋯H/H⋯O (15.0%), H⋯H (14.3%), Br⋯H/H⋯Br (14.2%), C⋯H/H⋯C (10.1%), F⋯H/H⋯F (7.9%), Br⋯Br (7.2%) and Br⋯C/C⋯Br (5.8%) contacts.

## Abstract 2

C—H⋯O and C—H⋯F hydrogen bonds link mol­ecules in the crystal into layers parallel to (011). The crystal packing is consolidated through C—Br⋯π and C—F⋯π inter­actions, as well as π–π stacking inter­actions.

## 1. Chemical context

Azo dyes are characterized by one or more azo groups *R*—N=N—*R*′, where *R* and *R*′ can be either alkyl, aryl or heterocyclic functional groups. Depending on the attached substituents, azo compounds have attracted attention because of their high synthetic potential for organic and inorganic chemistry and numerous useful properties. For example, azo dyes find applications in the design of functional materials attributed to smart hydrogen bonding, as self-assembled layers, photo-triggered structural switching, liquid crystals, ionophors, indicators, semiconductors, spectrophotometric reagents for determination of metal ions, catalysts, photoluminescent materials, optical recording media, spin-coating films and anti­microbial agents (Kopylovich *et al.*, 2012 ▸; Ma *et al.*, 2020 ▸, 2021 ▸; Mac Leod *et al.*, 2012 ▸; Viswanathan *et al.*, 2019 ▸). The azo-to-hydrazo tautomerism and *E*/*Z* isomerization properties of azo compounds are both crucial phenomena in the synthesis and design of new functional materials (Mahmudov *et al.*, 2012 ▸, 2013 ▸, 2020 ▸; Mizar *et al.*, 2012 ▸). Moreover, attachment of functional groups to the azo compounds acting as non-covalent donors or acceptors can be applied as a synthetic strategy for the improvement of the functional properties of this class of organic compounds (Gurbanov *et al.*, 2020*a* ▸,*b* ▸; Mahmoudi *et al.*, 2017 ▸, 2018 ▸; Shixaliyev *et al.*, 2013 ▸, 2014 ▸).

In the above context, we have attached F, Br and NO_2_ groups and aryl rings to the –N=N– moiety leading to a new azo compound, (*E*)-1-[2,2-di­bromo-1-(4-nitro­phen­yl)ethen­yl]-2-(4-fluoro­phen­yl)diazene, the mol­ecular and crystal structure of which along with a Hirshfeld surface analysis are reported here. 
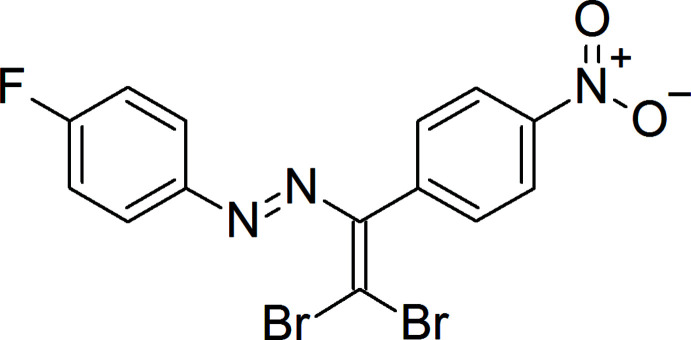


## 2. Structural commentary

**Figure 1:**
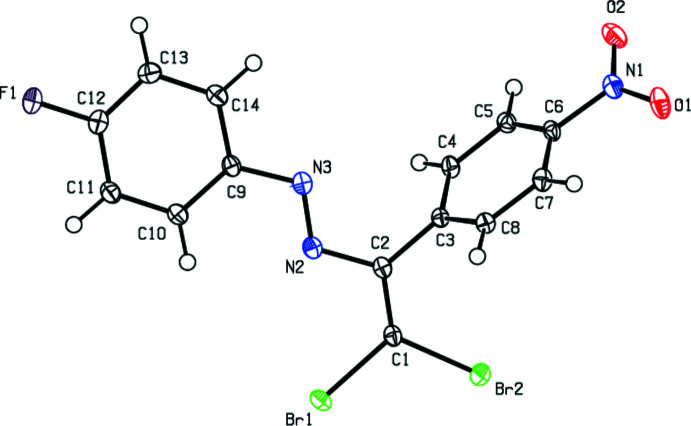
The title mol­ecule with the labelling scheme and displacement ellipsoids drawn at the 50% probability level.

The mol­ecular conformation of the title compound is not planar, as seen in Fig. 1 ▸, with the 4-fluoro­phenyl ring and the nitro-substituted phenyl ring subtending a dihedral angle of 64.37 (10)°. The C1=C2 double bond has a small twist, with the dihedral angle between atoms C1/Br1/Br2 and C2/C3/N2 being 3.99 (10)°, possibly to minimize steric repulsion between Br2 and H. The N3/N2/C2/C1/Br1/Br2 moiety subtends dihedral angles of 63.70 (8) and 1.39 (8)° with the C3–C8 and C9–C14 rings, respectively. The aromatic ring and olefin synthon in the mol­ecule are *trans*-configured with regard to the N=N double bond and are practically coplanar as revealed by the C2—N2=N3—C9 torsion angle of −178.63 (16)°. All of the bond lengths and angles in the title compound are similar to those for the related azo compounds reported in the *Database survey* section.

## 3. Supra­molecular features

**Figure 2:**
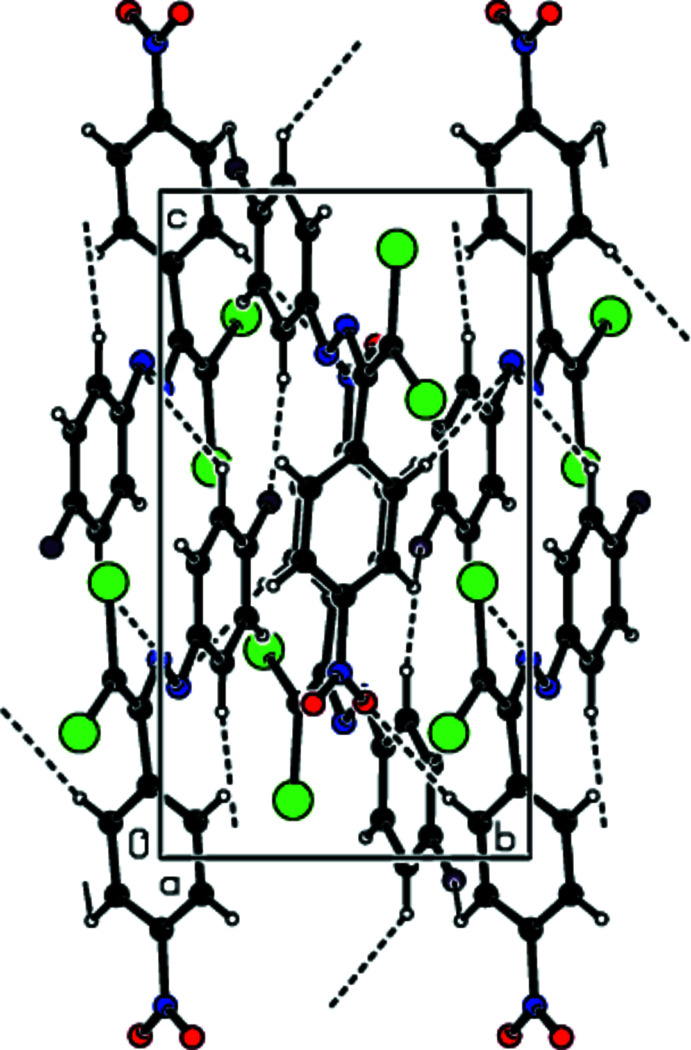
View down [100] of the C—H⋯O and C—H⋯F inter­actions (dashed lines) in the title compound.

**Figure 5:**
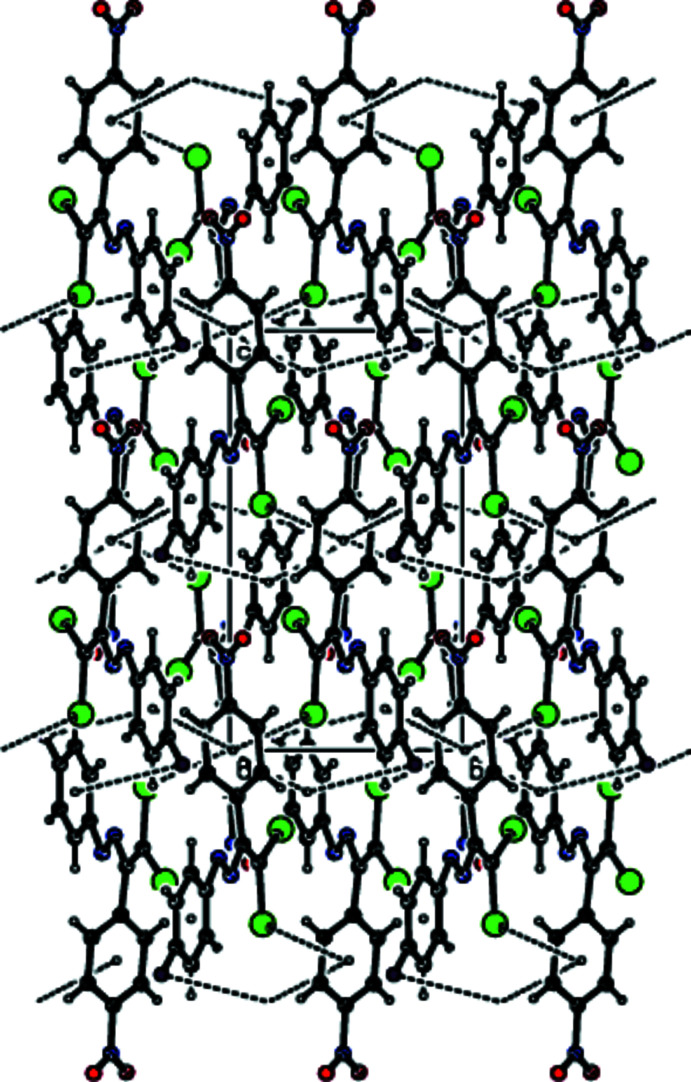
View down [100] of the title compound, showing the mol­ecular packing including C—Br⋯π and C—F⋯π inter­actions, as well as π–π inter­actions.

In the crystal, mol­ecules are linked by C—H⋯O and C—H⋯F hydrogen bonds into layers extending parallel to (011) (Table 1 ▸; Figs. 2 ▸–4 ▸ ▸). The crystal packing is consolidated by C—Br⋯π [Br1⋯*Cg*1 (*x*, 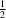 − *y*, − 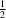 + *z*) = 3.6016 (9) Å, C1—Br1⋯*Cg*1 = 104.24 (7)°] and C—F⋯π [F1⋯*Cg*2 (1 − *x*, 1 − *y*, −*z*) = 3.5032 (17) Å, C12—F1⋯*Cg*2 = 92.53 (11)°] inter­actions, and weak π–π stacking [*Cg*1⋯*Cg*2 (*x*, 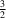 − *y*, 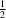 + *z*) = 4.0788 (12) Å, slippage = 1.776 Å], where *Cg*1 and *Cg*2 are the centroids of the C3–C8 and C9–C14 rings, respectively, (Figs. 5 ▸–7 ▸ ▸)].

## 4. Hirshfeld surface analysis

**Figure 8:**
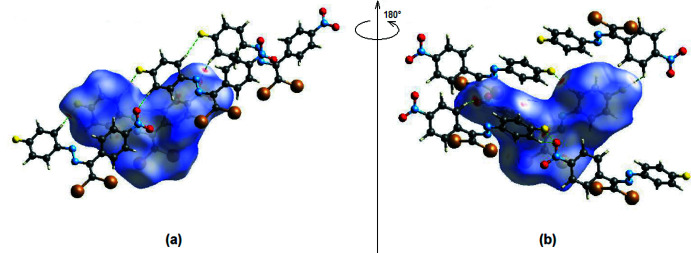
View of the three-dimensional Hirshfeld surface of the title compound plotted over *d*_norm_ in the range −0.1845 to 1.1463 a.u.

**Figure 9:**
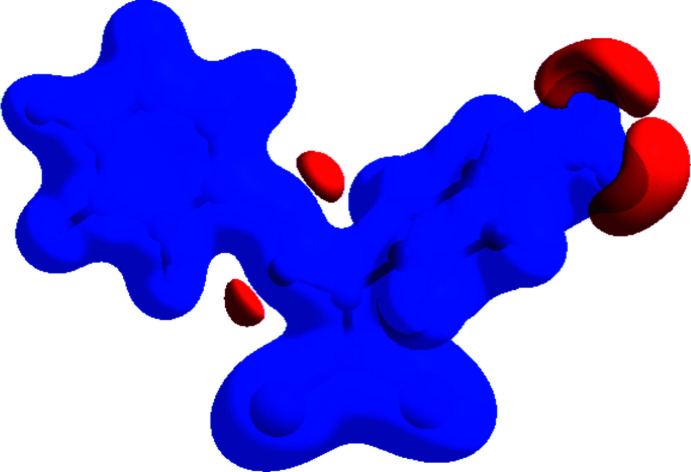
View of the three-dimensional Hirshfeld surface of the title complex plotted over electrostatic potential energy in the range −0.0500 to 0.0500 a.u. using the STO-3 G basis set at the Hartree–Fock level of theory. The hydrogen-bond donor and acceptor groups are viewed as blue and red regions, respectively around the atoms, corresponding to positive and negative potentials.

*Crystal Explorer 17.5* (Turner *et al.*, 2017 ▸) was used to perform a Hirshfeld surface analysis and to generate the corresponding two-dimensional fingerprint plots, with a standard resolution of the three-dimensional *d*_norm_ surfaces plotted over a fixed color scale of −0.1845 (red) to 1.1463 (blue) a.u. (Fig. 8 ▸). The red spots symbolize short contacts and negative *d*_norm_ values on the surface corresponding to the C—H⋯O and C—H⋯F hydrogen bonds described above (Table 1 ▸). The C4—H4⋯O2 and C11—H11⋯O2 inter­actions, which play a key role in the mol­ecular packing of the title compound, are responsible for the red spot that occurs around O2. The bright-red spots appearing near O2 and hydrogen atoms H4 and H11 indicate their roles as donor and/or acceptor groups in hydrogen bonding; they also appear as blue and red regions corres­ponding to positive and negative potentials on the Hirshfeld surface mapped over electrostatic potential (Spackman *et al.*, 2008 ▸) shown in Fig. 9 ▸.

**Figure 10:**
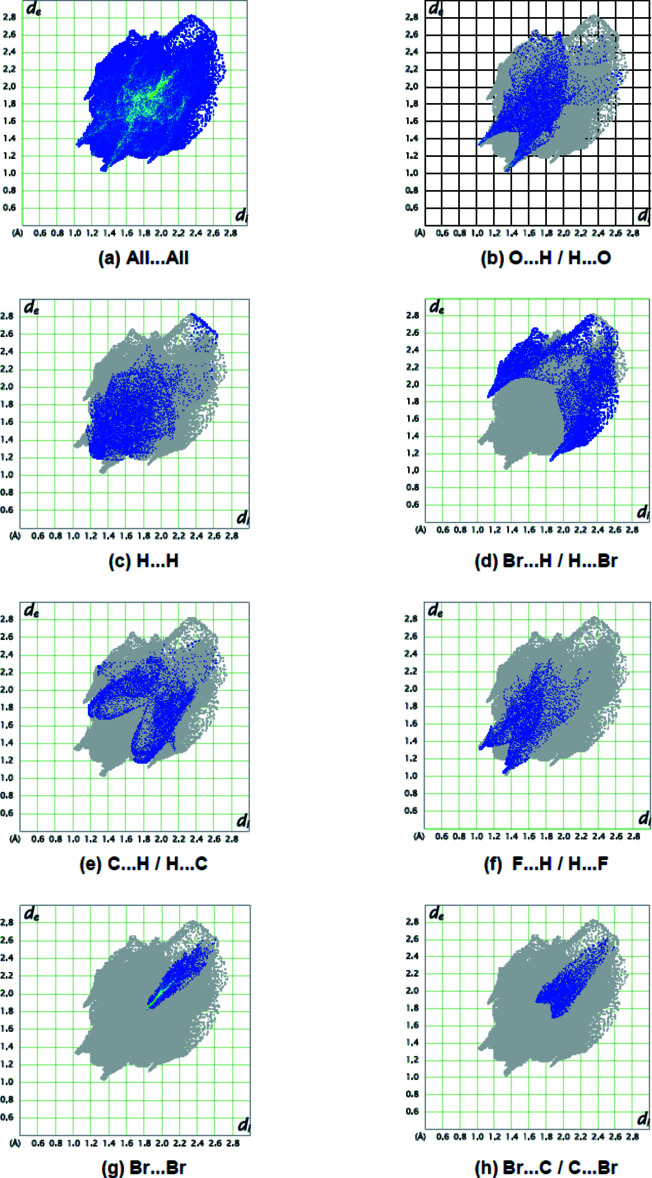
The full two-dimensional fingerprint plots for the title compound, showing (*a*) all inter­actions, and delineated into (*b*) O⋯H / H⋯O, (*c*) H⋯H, (*d*) Br⋯H / H⋯Br, (*e*) C⋯H / H⋯C, (*f*) F⋯H / H⋯F, (*g*) Br⋯Br and (*h*) Br⋯C / C⋯Br inter­actions. The *d*_i_ and *d*_e_ values are the closest inter­nal and external distances (in Å) from given points on the Hirshfeld surface.

The overall two-dimensional fingerprint plot for the title compound is given in Fig. 10 ▸ *a*, and those delineated into O⋯H/H⋯O, H⋯H, Br⋯H/H⋯Br, C⋯H/H⋯C, F⋯H/H⋯F, Br⋯Br and Br⋯C/C⋯Br contacts are shown in Fig.10*b*–*h*, while numerical details of the different contacts are given in Table 2 ▸. The percentage contributions to the Hirshfeld surfaces from the various inter­atomic contacts are compiled in Table 3 ▸. N⋯H/H⋯N, C⋯C, O⋯C/C⋯O, F⋯C/C⋯F, Br.·O/O⋯Br, N⋯C/C⋯N, N⋯O/O⋯N, N⋯N and F⋯F contacts contribute less than 5.7% to the Hirshfeld surface mapping and have little directional influence on the mol­ecular packing (Table 3 ▸).

## 5. Database survey

A search of the Cambridge Structural Database (CSD, Version 5.42, update of September 2021; Groom *et al.*, 2016 ▸) for the (*E*)-1-(2,2-di­chloro-1-phenyl­ethen­yl)-2-phenyl­diazene moiety resulted in 27 hits. Eight compounds are closely related to the title compound, *viz.* those with CSD refcodes GUPHIL (**I**) (Özkaraca *et al.*, 2020 ▸), HONBUK (**II**) (Akkurt *et al.*, 2019 ▸), HONBOE (**III**) (Akkurt *et al.*, 2019 ▸), HODQAV (**IV**) (Shikhaliyev *et al.*, 2019*a* ▸), XIZREG (**V**) (Atioğlu *et al.*, 2019 ▸), LEQXOX (**VI**) (Shikhaliyev *et al.*, 2018*a* ▸), LEQXIR (**VII**) (Shikhaliyev *et al.*, 2018*b* ▸) and PAXDOL (**VIII**) (Çelikesir *et al.*, 2022 ▸).

In the crystal of (**I**), mol­ecules are linked into inversion dimers *via* short halogen⋯halogen contacts [Cl1⋯Cl1 = 3.3763 (9) Å, C16—Cl1⋯Cl1 = 141.47 (7)°] compared to the van der Waals radius sum of 3.50 Å for two chlorine atoms. No other directional contacts could be identified, and the shortest aromatic ring centroid separation is greater than 5.25 Å. In the crystals of (**II**) and (**III**), mol­ecules are linked through weak *X*⋯Cl contacts [*X* = Cl for (**II**) and Br for (**III**)], C—H⋯Cl and C—Cl⋯π inter­actions into sheets lying parallel to (001). In the crystal of (**IV**), mol­ecules are stacked in columns parallel to [100] *via* weak C—H⋯Cl hydrogen bonds and face-to-face π–π stacking inter­actions. The crystal packing is further consolidated by short Cl⋯Cl contacts. In (**V**), mol­ecules are linked by C—H⋯O hydrogen bonds into zigzag chains running parallel to [001]. The crystal packing also features C—Cl⋯π, C—F⋯π and N—O⋯π inter­actions. In (**VI**), C—H⋯N and short Cl⋯Cl contacts are observed, and in (**VII**), C—H⋯N and C—H⋯O hydrogen bonds and short Cl⋯O contacts occur. In the crystal of (**VIII**), mol­ecules are linked into chains running parallel to [001] by C—H⋯O hydrogen bonds. The crystal packing is consolidated by C—F⋯π inter­actions and π–π stacking inter­actions, and short Br⋯O [2.9828 (13) Å] contacts are also observed.

## 6. Synthesis and crystallization

The title compound was synthesized according to a reported method (Akkurt et al., 2019 ▸; Atioğlu et al., 2019 ▸; Maharramov et al., 2018 ▸; Özkaraca et al., 2020 ▸; Shikhaliyev et al., 2018*a* ▸,*b* ▸, 2019*a* ▸,*b* ▸). A 20 ml screw-neck vial was charged with dimethyl sulfoxide (10 ml), (*E*)-1-(4-fluoro­phen­yl)-2-(4-nitro­benzyl­idene)hydrazine (1 mmol), tetra­methyl­ethylenedi­amine (295 mg, 2.5 mmol), CuCl (2 mg, 0.02 mmol) and CBr_4_ (4.5 mmol). After 1–3 h (until TLC analysis showed complete consumption of the corresponding Schiff base), the reaction mixture was poured into a 0.01 *M* HCl solution (100 ml, pH = 2–3), and extracted with di­chloro­methane (3 × 20 ml). The combined organic phase was washed with water (3 × 50 ml), brine (30 ml), dried over anhydrous Na_2_SO_4_ and concentrated *in vacuo* using a rotary evaporator. The residue was purified by column chromatography on silica gel using appropriate mixtures of hexane and di­chloro­methane (*v*/*v* 3/1–1/1). Light-orange solid (yield 52%); m.p. 377 K. Analysis calculated for C_14_H_8_Br_2_FN_3_O_2_ (M = 429.04): C 39.19, H 1.88, N 9.79; found: C 39.17, H 1.85, N 9.76%. ^1^H NMR (300MHz, CDCl_3_) δ 7.36–7.14 (8H, Ar–H). ^13^C NMR (75MHz, CDCl_3_) δ 164.35, 153.13, 152.46, 133.69, 133.24, 131.74, 127.98, 127.89, 127.75, 127.42, 119.07, 89.02. ESI–MS: *m*/*z*: 430.06 [*M* + H]^+^. Crystals suitable for X-ray analysis were obtained by slow evaporation of an ethanol solution.

## 7. Refinement

Crystal data, data collection and structure refinement details are summarized in Table 4 ▸. All H atoms were positioned geometrically [C—H = 0.95 Å] and refined using a riding model with *U*_iso_(H) = 1.2*U*_eq_(C). The maximum electron density in the final difference map is located 0.75 Å from atom Br1, while the minimum electron density is located 0.72 Å from Br2.

## Supplementary Material

Crystal structure: contains datablock(s) I. DOI: 10.1107/S2056989022004388/wm5642sup1.cif

Structure factors: contains datablock(s) I. DOI: 10.1107/S2056989022004388/wm5642Isup2.hkl

CCDC reference: 2168678

Additional supporting information:  crystallographic information; 3D view; checkCIF report

## Figures and Tables

**Figure 3:**
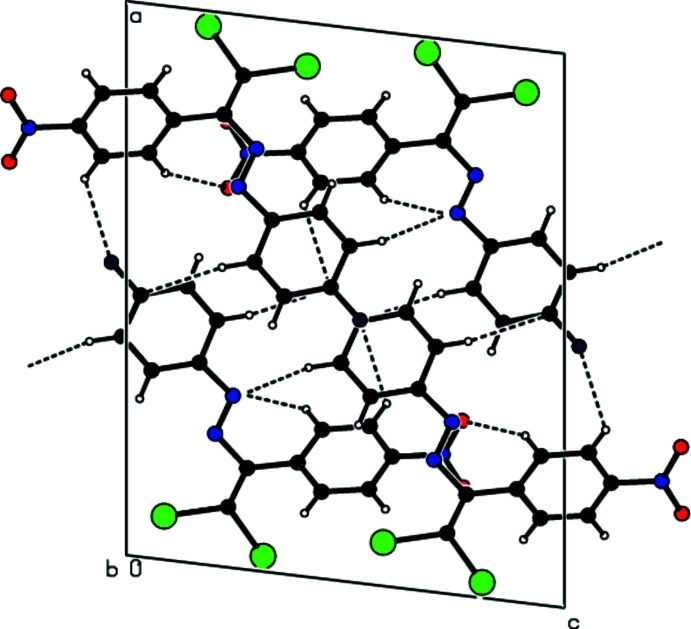
View down [010] of the C—H⋯O and C—H⋯F inter­actions (dashed lines) in the title compound.

**Figure 4:**
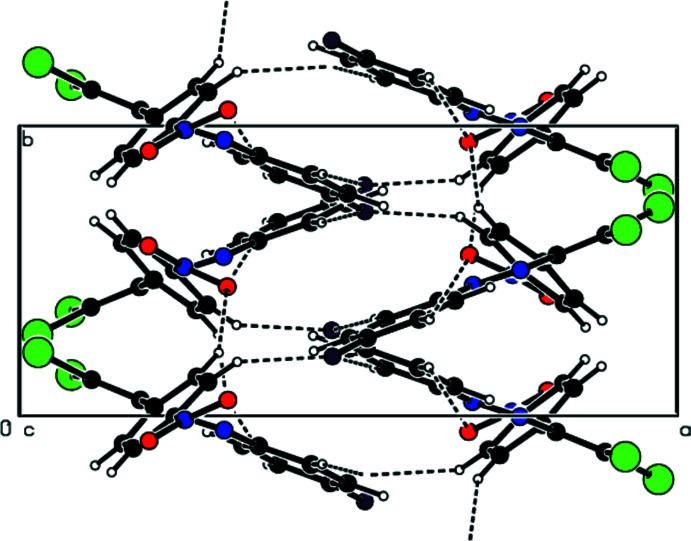
View down [001] of the C—H⋯O and C—H⋯F inter­actions (dashed lines) in the title compound.

**Figure 6:**
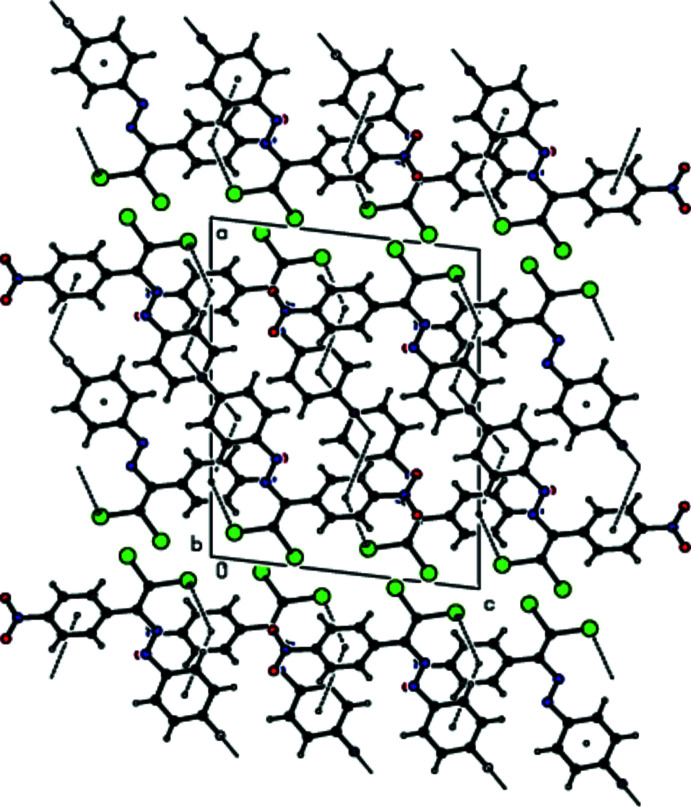
View down [010] of the title compound, showing the mol­ecular packing including C—Br⋯π and C—F⋯π inter­actions, as well as π–π inter­actions.

**Figure 7:**
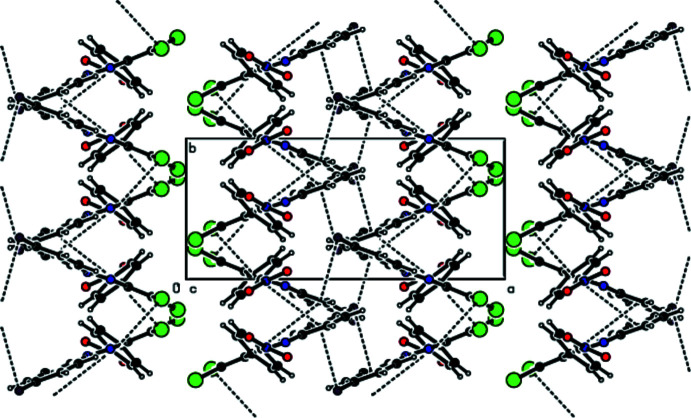
View down [001] of the title compound, showing the mol­ecular packing including C—Br⋯π and C—F⋯π inter­actions, as well as π–π inter­actions.

**Table 1:** Hydrogen-bond geometry (Å, °)

*D*—H⋯*A*	*D*—H	H⋯*A*	*D*⋯*A*	*D*—H⋯*A*
C4—H4⋯O2^i^	0.95	2.47	3.331 (3)	151
C5—H5⋯F1^ii^	0.95	2.54	3.150 (3)	122
C11—H11⋯O2^iii^	0.95	2.58	3.367 (3)	140
C14—H14⋯F1^iv^	0.95	2.49	3.427 (3)	169

Symmetry codes: (i) 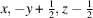 ; (ii) 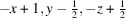 ; (iii) 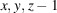 ; (iv) 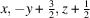 .

**Table 2:** Summary of short inter­atomic contacts (Å) in the title compound

Contact	Distance	Symmetry operation
C1⋯Br2	3.6060	−*x*, 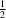 + *y*, 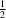 − *z*
Br1⋯Br1	3.7247	−*x*, 1 − *y*, −*z*
H4⋯O2	2.47	*x*, 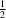 − *y*, − 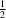 + *z*
H7⋯Br2	3.08	−*x*, 1 − *y*, 1 − *z*
F1⋯H5	2.54	1 − *x*, 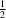 + *y*, 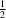 − *z*
C12⋯F1	3.3310	1 − *x*, 2 − *y*, −*z*
H14⋯F1	2.49	*x*, 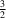 − *y*, 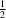 + *z*
O2⋯H11	2.58	*x*, *y*, 1 + *z*
H13⋯O2	2.69	1 − *x*, 1 − *y*, 1 − *z*
C12⋯C12	3.5050	1 − *x*, 1 − *y*, −*z*

**Table 3:** Percentage contributions of inter­atomic contacts to the Hirshfeld surface for the title compound

Contact	Percentage contribution
O⋯H/H⋯O	15.0
H⋯H	14.3
Br⋯H/H⋯Br	14.2
C⋯H/H⋯C	10.1
F⋯H/H⋯F	7.9
Br⋯Br	7.2
Br.·C/C⋯Br	5.8
N⋯H/H⋯N	5.7
C⋯C	4.2
O⋯C/C⋯O	4.0
F⋯C/C⋯F	3.1
Br.·O/O⋯Br	2.7
N⋯C/C⋯N	2.1
N⋯O/O⋯N	2.0
N⋯N	1.0
F⋯F	0.8

**Table 4:** Experimental details

Crystal data	
Chemical formula	C_14_H_8_Br_2_FN_3_O_2_
*M*_r_	429.05
Crystal system, space group	Monoclinic, *P*2_1_/*c*
Temperature (K)	100
*a*, *b*, *c* (Å)	16.0658 (2), 7.0329 (1), 12.7934 (2)
β (°)	96.8470 (6)
*V* (Å^3^)	1435.21 (4)
*Z*	4
Radiation type	Mo *K*α
μ (mm^−1^)	5.67
Crystal size (mm)	0.37 × 0.21 × 0.08

Data collection	
Diffractometer	Bruker AXS D8 QUEST, Photon III detector
Absorption correction	Multi-scan (*SADABS*; Krause *et al.*, 2015 ▸)
*T*_min_, *T*_max_	0.415, 0.747
No. of measured, independent and observed [*I* > 2σ(*I*)] reflections	44165, 4177, 3809
*R*_int_	0.102
(sin θ/λ)_max_ (Å^−1^)	0.703

Refinement	
*R*[*F* ^2^ > 2σ(*F* ^2^)], *wR*(*F* ^2^), *S*	0.034, 0.096, 1.05
No. of reflections	4177
No. of parameters	199
H-atom treatment	H-atom parameters constrained
Δρ_max_, Δρ_min_ (e Å^−3^)	2.00, −1.04

Computer programs: *APEX3* and *SAINT* (Bruker, 2018 ▸), *SHELXT* (Sheldrick, 2015*a* ▸), *SHELXL2018* (Sheldrick, 2015*b* ▸), *ORTEP-3 for Windows* (Farrugia, 2012 ▸) and *PLATON* (Spek, 2020 ▸).

## supplementary crystallographic information

### Crystal data

**Table:**

C_14_H_8_Br_2_FN_3_O_2_	*F*(000) = 832
*M**_r_* = 429.05	*D*_x_ = 1.986 Mg m^−^^3^
Monoclinic, *P*2_1_/*c*	Mo *K*α radiation, λ = 0.71073 Å
*a* = 16.0658 (2) Å	Cell parameters from 9926 reflections
*b* = 7.0329 (1) Å	θ = 3.2–33.2°
*c* = 12.7934 (2) Å	µ = 5.67 mm^−^^1^
β = 96.8470 (6)°	*T* = 100 K
*V* = 1435.21 (4) Å^3^	Plate, light red
*Z* = 4	0.37 × 0.21 × 0.08 mm

### Data collection

**Table:**

Bruker AXS D8 QUEST, Photon III detector diffractometer	4177 independent reflections
Radiation source: fine-focus sealed X-Ray tube	3809 reflections with *I* > 2σ(*I*)
Graphite monochromator	*R*_int_ = 0.102
Detector resolution: 7.31 pixels mm^-1^	θ_max_ = 30.0°, θ_min_ = 2.6°
φ and ω shutterless scans	*h* = −22→22
Absorption correction: multi-scan (SADABS; Krause *et al.*, 2015)	*k* = −9→9
*T*_min_ = 0.415, *T*_max_ = 0.747	*l* = −17→17
44165 measured reflections	

### Refinement

**Table:**

Refinement on *F*^2^	Primary atom site location: structure-invariant direct methods
Least-squares matrix: full	Secondary atom site location: difference Fourier map
*R*[*F*^2^ > 2σ(*F*^2^)] = 0.034	Hydrogen site location: inferred from neighbouring sites
*wR*(*F*^2^) = 0.096	H-atom parameters constrained
*S* = 1.05	*w* = 1/[σ^2^(*F*_o_^2^) + (0.0647*P*)^2^ + 0.5442*P*] where *P* = (*F*_o_^2^ + 2*F*_c_^2^)/3
4177 reflections	(Δ/σ)_max_ < 0.001
199 parameters	Δρ_max_ = 2.00 e Å^−^^3^
0 restraints	Δρ_min_ = −1.04 e Å^−^^3^

### Special details

**Table:**

Geometry. All esds (except the esd in the dihedral angle between two l.s. planes) are estimated using the full covariance matrix. The cell esds are taken into account individually in the estimation of esds in distances, angles and torsion angles; correlations between esds in cell parameters are only used when they are defined by crystal symmetry. An approximate (isotropic) treatment of cell esds is used for estimating esds involving l.s. planes.
Refinement. Refinement of F^2^ against ALL reflections. The weighted R-factor wR and goodness of fit S are based on F^2^, conventional R-factors R are based on F, with F set to zero for negative F^2^. The threshold expression of F^2^ > 2sigma(F^2^) is used only for calculating R-factors(gt) etc. and is not relevant to the choice of reflections for refinement. R-factors based on F^2^ are statistically about twice as large as those based on F, and R- factors based on ALL data will be even larger.

### Fractional atomic coordinates and isotropic or equivalent isotropic displacement parameters (Å^2^)

**Table:**

	*x*	*y*	*z*	*U*_iso_*/*U*_eq_	
Br1	0.07857 (2)	0.35922 (3)	0.08663 (2)	0.01718 (8)	
Br2	0.02778 (2)	0.28084 (3)	0.31239 (2)	0.02246 (8)	
C1	0.10911 (13)	0.3752 (3)	0.23267 (15)	0.0143 (3)	
C2	0.18336 (12)	0.4450 (3)	0.27612 (15)	0.0135 (3)	
C3	0.20411 (12)	0.4693 (3)	0.39184 (15)	0.0131 (3)	
C4	0.26892 (13)	0.3653 (3)	0.44734 (16)	0.0151 (4)	
H4	0.301935	0.282209	0.410651	0.018*	
C5	0.28537 (13)	0.3827 (3)	0.55606 (16)	0.0148 (3)	
H5	0.329792	0.313645	0.594394	0.018*	
C6	0.23537 (13)	0.5033 (3)	0.60701 (15)	0.0141 (3)	
C7	0.17169 (13)	0.6115 (3)	0.55419 (16)	0.0158 (4)	
H7	0.139047	0.694455	0.591491	0.019*	
C8	0.15669 (12)	0.5958 (3)	0.44533 (15)	0.0143 (3)	
H8	0.114268	0.670850	0.407127	0.017*	
N1	0.24975 (12)	0.5135 (2)	0.72236 (14)	0.0178 (3)	
O1	0.19544 (12)	0.5861 (2)	0.76893 (13)	0.0259 (3)	
O2	0.31534 (11)	0.4454 (2)	0.76654 (12)	0.0245 (3)	
N2	0.23866 (11)	0.4967 (2)	0.20300 (13)	0.0153 (3)	
N3	0.31074 (11)	0.5492 (3)	0.24360 (14)	0.0165 (3)	
C9	0.36346 (12)	0.6039 (3)	0.16716 (15)	0.0144 (3)	
C10	0.33917 (13)	0.6020 (3)	0.05795 (16)	0.0158 (4)	
H10	0.285307	0.556049	0.030764	0.019*	
C11	0.39360 (14)	0.6669 (3)	−0.00982 (16)	0.0177 (4)	
H11	0.377788	0.667540	−0.083780	0.021*	
C12	0.47214 (14)	0.7314 (3)	0.03294 (17)	0.0185 (4)	
C13	0.49862 (14)	0.7325 (3)	0.13950 (18)	0.0203 (4)	
H13	0.553030	0.776322	0.165862	0.024*	
C14	0.44330 (14)	0.6675 (3)	0.20716 (17)	0.0186 (4)	
H14	0.459864	0.666418	0.280965	0.022*	
F1	0.52527 (9)	0.7950 (2)	−0.03383 (11)	0.0261 (3)	

### Atomic displacement parameters (Å^2^)

**Table:**

	*U*^11^	*U*^22^	*U*^33^	*U*^12^	*U*^13^	*U*^23^
Br1	0.01901 (12)	0.01931 (12)	0.01257 (11)	0.00170 (7)	−0.00084 (8)	−0.00163 (6)
Br2	0.02126 (13)	0.02844 (14)	0.01798 (12)	−0.00940 (8)	0.00360 (8)	0.00113 (8)
C1	0.0179 (9)	0.0133 (8)	0.0121 (8)	−0.0006 (7)	0.0030 (7)	0.0007 (6)
C2	0.0173 (8)	0.0111 (8)	0.0124 (8)	0.0016 (6)	0.0022 (6)	0.0007 (6)
C3	0.0144 (8)	0.0131 (8)	0.0118 (8)	−0.0003 (6)	0.0022 (6)	0.0005 (6)
C4	0.0176 (9)	0.0159 (9)	0.0124 (8)	0.0025 (7)	0.0038 (7)	0.0002 (6)
C5	0.0165 (8)	0.0144 (8)	0.0134 (8)	0.0000 (7)	0.0013 (7)	0.0020 (7)
C6	0.0196 (9)	0.0124 (8)	0.0105 (8)	−0.0044 (7)	0.0027 (7)	−0.0002 (6)
C7	0.0197 (9)	0.0124 (8)	0.0159 (9)	0.0001 (7)	0.0052 (7)	−0.0017 (7)
C8	0.0156 (8)	0.0133 (8)	0.0142 (8)	0.0016 (7)	0.0023 (7)	0.0000 (7)
N1	0.0269 (9)	0.0122 (7)	0.0147 (8)	−0.0054 (6)	0.0037 (6)	−0.0008 (6)
O1	0.0394 (9)	0.0243 (8)	0.0159 (7)	0.0010 (7)	0.0107 (7)	−0.0038 (6)
O2	0.0325 (9)	0.0245 (8)	0.0150 (7)	−0.0041 (7)	−0.0029 (6)	0.0020 (6)
N2	0.0182 (8)	0.0141 (7)	0.0139 (7)	0.0018 (6)	0.0026 (6)	0.0002 (6)
N3	0.0192 (8)	0.0158 (8)	0.0148 (7)	0.0010 (6)	0.0034 (6)	0.0005 (6)
C9	0.0172 (9)	0.0133 (8)	0.0128 (8)	0.0019 (7)	0.0026 (7)	−0.0002 (7)
C10	0.0179 (9)	0.0154 (8)	0.0140 (8)	0.0004 (7)	0.0012 (7)	−0.0010 (7)
C11	0.0212 (9)	0.0184 (9)	0.0132 (8)	−0.0013 (8)	0.0015 (7)	−0.0007 (7)
C12	0.0194 (9)	0.0183 (9)	0.0188 (9)	−0.0006 (7)	0.0062 (8)	0.0009 (7)
C13	0.0168 (9)	0.0232 (10)	0.0205 (10)	−0.0025 (8)	0.0012 (8)	−0.0018 (8)
C14	0.0198 (9)	0.0217 (9)	0.0140 (9)	−0.0006 (8)	0.0004 (7)	−0.0017 (7)
F1	0.0239 (7)	0.0354 (8)	0.0202 (6)	−0.0064 (6)	0.0081 (5)	0.0037 (6)

### Geometric parameters (Å, º)

**Table:**

Br1—C1	1.878 (2)	N1—O1	1.225 (2)
Br2—C1	1.872 (2)	N1—O2	1.232 (3)
C1—C2	1.347 (3)	N2—N3	1.266 (2)
C2—N2	1.412 (3)	N3—C9	1.421 (3)
C2—C3	1.488 (3)	C9—C14	1.397 (3)
C3—C4	1.395 (3)	C9—C10	1.405 (3)
C3—C8	1.402 (3)	C10—C11	1.380 (3)
C4—C5	1.390 (3)	C10—H10	0.9500
C4—H4	0.9500	C11—C12	1.390 (3)
C5—C6	1.384 (3)	C11—H11	0.9500
C5—H5	0.9500	C12—F1	1.354 (2)
C6—C7	1.385 (3)	C12—C13	1.379 (3)
C6—N1	1.468 (2)	C13—C14	1.390 (3)
C7—C8	1.389 (3)	C13—H13	0.9500
C7—H7	0.9500	C14—H14	0.9500
C8—H8	0.9500		
			
C2—C1—Br2	123.06 (15)	O1—N1—O2	123.97 (19)
C2—C1—Br1	123.08 (15)	O1—N1—C6	118.25 (18)
Br2—C1—Br1	113.85 (10)	O2—N1—C6	117.77 (17)
C1—C2—N2	114.61 (17)	N3—N2—C2	114.84 (17)
C1—C2—C3	122.32 (17)	N2—N3—C9	112.81 (17)
N2—C2—C3	123.05 (17)	C14—C9—C10	120.10 (19)
C4—C3—C8	120.02 (18)	C14—C9—N3	115.55 (18)
C4—C3—C2	120.73 (17)	C10—C9—N3	124.33 (18)
C8—C3—C2	119.23 (17)	C11—C10—C9	119.98 (19)
C5—C4—C3	120.28 (18)	C11—C10—H10	120.0
C5—C4—H4	119.9	C9—C10—H10	120.0
C3—C4—H4	119.9	C10—C11—C12	118.29 (19)
C6—C5—C4	118.27 (18)	C10—C11—H11	120.9
C6—C5—H5	120.9	C12—C11—H11	120.9
C4—C5—H5	120.9	F1—C12—C13	118.6 (2)
C5—C6—C7	122.93 (18)	F1—C12—C11	118.07 (19)
C5—C6—N1	118.21 (18)	C13—C12—C11	123.3 (2)
C7—C6—N1	118.85 (17)	C12—C13—C14	118.0 (2)
C6—C7—C8	118.38 (18)	C12—C13—H13	121.0
C6—C7—H7	120.8	C14—C13—H13	121.0
C8—C7—H7	120.8	C13—C14—C9	120.3 (2)
C7—C8—C3	120.04 (18)	C13—C14—H14	119.8
C7—C8—H8	120.0	C9—C14—H14	119.8
C3—C8—H8	120.0		
			
Br2—C1—C2—N2	175.66 (13)	C7—C6—N1—O1	14.3 (3)
Br1—C1—C2—N2	−3.1 (3)	C5—C6—N1—O2	14.4 (3)
Br2—C1—C2—C3	−5.6 (3)	C7—C6—N1—O2	−166.82 (18)
Br1—C1—C2—C3	175.56 (14)	C1—C2—N2—N3	−175.03 (18)
C1—C2—C3—C4	115.8 (2)	C3—C2—N2—N3	6.3 (3)
N2—C2—C3—C4	−65.7 (3)	C2—N2—N3—C9	−178.63 (16)
C1—C2—C3—C8	−63.1 (3)	N2—N3—C9—C14	178.57 (18)
N2—C2—C3—C8	115.5 (2)	N2—N3—C9—C10	0.4 (3)
C8—C3—C4—C5	1.7 (3)	C14—C9—C10—C11	−1.4 (3)
C2—C3—C4—C5	−177.12 (18)	N3—C9—C10—C11	176.7 (2)
C3—C4—C5—C6	0.8 (3)	C9—C10—C11—C12	0.6 (3)
C4—C5—C6—C7	−2.1 (3)	C10—C11—C12—F1	−179.95 (19)
C4—C5—C6—N1	176.62 (18)	C10—C11—C12—C13	0.5 (3)
C5—C6—C7—C8	1.0 (3)	F1—C12—C13—C14	179.7 (2)
N1—C6—C7—C8	−177.77 (17)	C11—C12—C13—C14	−0.7 (3)
C6—C7—C8—C3	1.5 (3)	C12—C13—C14—C9	−0.1 (3)
C4—C3—C8—C7	−2.9 (3)	C10—C9—C14—C13	1.2 (3)
C2—C3—C8—C7	175.96 (18)	N3—C9—C14—C13	−177.1 (2)
C5—C6—N1—O1	−164.51 (19)		

### Hydrogen-bond geometry (Å, º)

**Table:**

*D*—H···*A*	*D*—H	H···*A*	*D*···*A*	*D*—H···*A*
C4—H4···O2^i^	0.95	2.47	3.331 (3)	151
C5—H5···F1^ii^	0.95	2.54	3.150 (3)	122
C11—H11···O2^iii^	0.95	2.58	3.367 (3)	140
C14—H14···F1^iv^	0.95	2.49	3.427 (3)	169

Symmetry codes: (i) *x*, −*y*+1/2, *z*−1/2; (ii) −*x*+1, *y*−1/2, −*z*+1/2; (iii) *x*, *y*, *z*−1; (iv) *x*, −*y*+3/2, *z*+1/2.
